# Associations of modifiable and non-modifiable risk factors with longitudinal white matter hyperintensities, amyloid-β and tau - a prospective cohort study

**DOI:** 10.1016/j.tjpad.2025.100448

**Published:** 2026-01-01

**Authors:** Isabelle Glans, Niklas Mattsson-Carlgren, Olof Strandberg, Erik Stomrud, Rik Ossenkoppele, Danielle van Westen, Nicola Spotorno, Oskar Hansson, Sebastian Palmqvist

**Affiliations:** a Clinical Memory Research Unit, Department of Clinical Sciences, Malmö, Lund University, Sweden; b Memory Clinic, Skåne University Hospital, Malmö, Sweden; c Alzheimer Center Amsterdam, Neurology, Vrije Universiteit Amsterdam, Amsterdam UMC, Amsterdam, the Netherlands; d Amsterdam Neuroscience, Neurodegeneration, Amsterdam, the Netherlands; e Diagnostic Radiology, Department of Clinical Sciences Lund, Lund University, Sweden; f Image and Function, Skåne University Hospital, Lund, Sweden

**Keywords:** Risk factors, Dementia, Aβ, Tau, Vascular disease, White-matter hyperintensities, Prevention

## Abstract

**Background:**

The global prevalence of dementia is rapidly expanding and is expected to triple by 2050. Approximately 45 % of dementia cases are estimated to be attributable to potentially modifiable risk factors. Identifying how these factors contribute to specific brain pathologies may improve strategies to reduce dementia incidence.

**Objectives, Design, Setting:**

The aim of this study was to identify both non-modifiable and modifiable risk factors associated with longitudinal changes in white matter hyperintensities (WMH), amyloid-beta (Aβ) and tau. Data were acquired in the prospective observational Swedish BioFINDER-2 study between May 2017-January 2025. All participants underwent clinical assessments, questionnaires and at least two magnetic resonance imaging (MRI), Aβ Positron Emission Tomography (PET) and tau PET scans, respectively. Mixed-effects models were used to assess the associations between non-modifiable and modifiable risk factors and WMH (MRI), Aβ (PET) and tau (PET).

**Participants:**

A total of 494 cognitively unimpaired participants were included, of whom 365 were amyloid-negative (CU Aβ−) and 129 were amyloid-positive (CU Aβ+).

**Measurements and main outcomes:**

Non-modifiable (age, apolipoprotein E *(APOE)* ɛ4 genotype and sex) and modifiable risk factors (co-morbidities at baseline, such as hypertension and cardiovascular disease, BMI, and sleep duration) were analyzed with mixed-effects models, adjusted for age and sex, to predict longitudinal measurements of WMH, Aβ and tau.

**Results:**

Mean age was 64.8 (SD 13.3) years and mean follow-up was 3.9 (SD 1.5) years. Predictors represent baseline data, both predictors and outcomes are on standardized scales. Linear mixed-effects models, adjusted for age and sex, showed that higher blood pressure (β = 0.02, 95 % CI :0.01–0.02), presence of hyperlipidemia (β = 0.03, 0.01–0.05), ischemic heart disease (β = 0.06, 0.03–0.09), smoking (β = 0.02, 0.00–0.03) and lower education (β = -0.01, -0.02– -0.01) were associated with a longitudinal increase in WMH. Presence of the *APOE* ε4 allele was linked to faster Aβ accumulation (β = 0.03, 0.02–0.04) and tau (β = 0.01, 0.00–0.03), but only to Aβ among Aβ+ positive participants. Higher depression score (β = 0.01, 0.00–0.01) and diabetes (β = 0.02, 0.00–0.04) were associated with faster Aβ accumulation. Lower BMI was associated with faster accumulation of tau (β = -0.01, -0.02– -0.01).

**Conclusions:**

Modifiable risk factors of future dementia primarily affect accumulation of cerebral vascular pathology, although lower BMI was associated with tau accumulation and diabetes with Aβ accumulation.

## 1 Introduction

The global prevalence of dementia is expected to triple by 2050, resulting in a substantial burden on affected individuals, caregivers, and healthcare systems. Since an estimated 45 % of dementia cases are linked to potentially modifiable risk factors, there exists a significant opportunity for preventive interventions [1]. A key goal in reducing dementia incidence involves understanding the mechanisms through which these risk factors drive the neuropathological processes underlying dementia and to develop effective intervention strategies aimed at reducing their impact.

The most common causes of major neurocognitive disorder, or dementia, are Alzheimer’s disease (AD) and vascular pathology, and these often coexist [2,3]. Although vascular and AD pathologies are distinct entities they can exacerbate each other, lowering the threshold for symptom onset at a given burden of AD pathology [4]. AD pathology, i.e., amyloid β plaques (Aβ) and tau neurofibrillary tangles, is central to the amyloid cascade hypothesis of AD [5] and their accumulation in the brain can be visualized using Positron Emission Tomography (PET) [6].

Vascular cognitive impairment is the second most common cause of neurocognitive disorders after AD. Cognitive impairment caused by cerebrovascular disease can be of either ischemic or hemorrhagic type, and the ischemic type is most commonly caused by small vessel disease [7]. White matter hyperintensities (WMH) are the most common neuroimaging feature of small vessel disease and can be detected on magnetic resonance imaging (MRI) [8].

While age is a common risk factor for vascular and AD pathology, small vessel disease has consistently been associated with metabolic risk factors - such as high blood pressure, diabetes [9,10], and smoking [11]. Obesity though presents a paradox; while midlife obesity increases the risk of vascular dementia, lower body mass index (BMI) has been associated with a higher likelihood of AD biomarker positivity [12,13]. This raises the question of whether it is a cause, a consequence, or both. Also, cognitive impairment in overweight individuals may be more likely to result from heterogeneous pathophysiology due to higher prevalence of metabolic syndrome, hypertension and diabetes [13].

Risk factors for Aβ pathology include apolipoprotein E *(APOE)* ɛ4 genotype, whereas the abovementioned vascular risk factors have not been shown to influence Aβ pathology [12,14] although frequently highlighted as risk factors of AD [15]. Previous studies have shown that tau accumulates more rapidly in women than in men [16,17]. However, studies investigating how modifiable risk factors contribute to the accumulation of Aβ and tau measured by PET remain scarce.

The aim of this study was to examine the effect of both modifiable and non-modifiable risk factors on longitudinal accumulation of WMH (MRI), Aβ (PET) and tau (PET). We hypothesized that small vessel disease represents the pathology, among these, that is most susceptible to modification. This investigation was conducted in a prospective study cohort of 494 individuals, who were followed for a mean duration of 3.9 years (range: 1.5–6.5 years).

## 2 Methods

### 2.1 Participants

We included participants from the prospective Swedish BioFINDER-2 study (NCT03174938). The study has been described extensively elsewhere and additional information can be found at https://biofinder.se/two/ [18]. All participants underwent detailed assessments, including cerebrospinal fluid (CSF) analysis, PET imaging, MRI, and clinical and cognitive evaluations. Inclusion criteria for the present study were 1) cognitively unimpaired (CU; either cognitively healthy control or subjective cognitive decline), 2) at least two of each of the following examinations: MRI, Aβ PET and tau PET and 3) a complete dataset for all studied predictors. See the eMethods in the Supplement for details on diagnostic and inclusion criteria. Presence of cognitive impairment was ruled out using a large neuropsychological battery as previously described [19]. Individuals were cognitively unimpaired at baseline to minimize the risk of reverse causation when examining the risk factors. This resulted in a population of 494 participants with complete data for all predictors and outcomes. Data was collected between May 2017 and January 2025. Participants were recruited at the Skåne University Hospital and the hospital of Ängelholm, Sweden. The study was approved by the regional Ethical Review Board at Lund University, the Swedish Medical Products Agency and local radiation committee at Skåne University Hospital. All participants gave written informed consent prior to participation in the study.

### 2.2 Predictors

All participants completed baseline questionnaires covering demographic factors such as years of education and pre-existing conditions at baseline, including hypertension, diabetes, hyperlipidemia, stroke/TIA and other diseases. Biological sex from personal identity number was used to define gender. *APOE* genotype analyzed from blood was divided into two groups: presence or absence of at least one ɛ4 allele. Absence was used as reference group in the statistical models, since carrying an ɛ4 allele increases the risk of developing AD [20]. The questionnaire also included self-reported information on alcohol consumption (measured in standard drinks/week using visual aids, see supplementary eMethods), current medications, and smoking status (never, former, or current). Current medication and previous diseases were validated and classified by medical doctors at the Memory Clinic, Skåne University Hospital, Malmö. Blood pressure was measured in a supine position at each visit after 15 min of rest. Body mass index (BMI) was calculated as weight divided by height squared (kg/m²) and was measured at each visit. Participants also completed the Hospital Anxiety and Depression scale (HADS), with sub-scores for depression and anxiety used as separate predictors. Sleep duration was derived from the Sleep Scale from the Medical Outcomes study, covering the last 4 weeks [21]. Continuous variables were converted to z-scores to facilitate comparisons of estimates. All predictors represent baseline measurements or estimates. All predictor baseline data were complete.

### 2.3 CSF

CSF samples were collected at baseline. Lumbar puncture and CSF handling followed a structured protocol [22]. Levels of Aβ42 and Aβ40 were measured using Elecsys immunoassays [23]. CSF Aβ positivity (Aβ+) was defined as CSF Aβ42/Aβ40 of <0.08 [24].

### 2.4 MRI and PET imaging

MRI and PET imaging was performed every second year, except for individuals <40 years (*n* = 18) where PET imaging was performed every fourth year. T1-weighted MRI sequences were acquired on a 3 Tesla MAGNETOM Siemens Prisma scanner. MRI was performed at baseline and repeated close to follow-up PET scans. WMH were automatically segmented on T2-weighted FLAIR sequences using the lesion prediction algorithm implemented in the lesion segmentation toolbox (https://www.applied-statistics.de/lst.html) resulting in a total lesion volume in [mL] for each individual. Intracranial volumes were estimated as part of T1-weighted image processing in FreeSurfer (v6; https://surfer.nmr.mgh.harvard.edu/) [25]. For visual quantification of WMH load, the Fazekas rating scale (0–3) was used, evaluated by an experienced neuroradiologist. This was not used in statistical analysis but only for presentation purposes in Table 1 [26]. Aβ PET imaging was performed on the same platform as tau PET 90–110 min after the injection of ∼185 MBq [^18^F]Flutemetamol. Standardized uptake value ratios (SUVr) were calculated with cerebellum as reference region. Tau PET images were acquired on the digital GE Discovery MI scanners using [^18^F]RO948. Images were acquired 70–90 min after injection of ∼370 MBq [^18^F]RO948. SUVr images were created using the inferior cerebellar cortex as the reference region [27]. Tau-PET SUVr was calculated in a temporal meta-ROI consisting of the entorhinal cortex, amygdala, parahippocampal gyrus, fusiform gyrus, and inferior and middle temporal gyrus, corresponding to Braak stages I-IV. Full PET details have been previously described [28]. The Boston criteria version 2.0 for cerebral amyloid angiopathy (CAA) was used for evaluating presence of CAA among Aβ + individuals [29].

**Table 1:** Baseline demographics.

	CU Aβ- (*n* = 365)	CU Aβ+ (*n* = 129)	All (*n* = 494)	*p*-value
Age (years)	62.5 (13.9)	71.2 (8.9)	64.8 (13.3)	*<0.001*
Education (years)	13.2 (3.6)	12.9 (3.9)	13.1 (3.6)	*<0.001*
*APOE* genotype (n,%)				
ε4-carrier	144 (39.5)	99 (76.7)	243 (49.2)	*<0.001*
Alcohol consumption (n,%)				
0 standard drinks/week	117 (32.1)	43 (33.3)	160 (32.4)	0.777
1–9 standard drinks/week	228 (62.5)	80 (62.0)	308 (62.3)	0.777
>10 standard drinks/week	20 (5.5)	6 (4.7)	26 (5.3)	0.777
Blood pressure (systolic)	141 (19.2)	146 (19.4)	143 (19.3)	*<0.001*
Body mass index^a^	26.9 (4.3)	25.8 (3.9)	26.6 (4.3)	*<0.001*
Diabetes (n,%)	32 (8.9)	16 (12.6)	48 (9.7)	*<0.05*
Fazekas Rating Scale^b^ (n,%)				
0	67 (18.4)	10 (7.8)	77 (15.6)	*<0.001*
1	200 (54.8)	63 (48.8)	263 (53.2)	0.36
2	59 (16.2)	27 (20.9)	86 (17.4)	*<0.001*
3	13 (3.6)	10 (14.7)	23 (4.7)	*<0.001*
Hyperlipidemia (n,%)	39 (10.8)	21 (16.5)	60 (12.1)	*<0.05*
HADS, depression score	2.1 (2.6)	2.2 (2.2)	2.1 (2.5)	*<0.001*
Living alone (n,%)	100 (27.4)	44 (34.1)	144 (29.1)	*<0.001*
Hypertensive or cardioprotective medications (n,%)	125 (34.2)	58 (45.0)	183 (37.0)	*<0.001*
Ischemic heart disease (n,%)	22 (6.0)	7 (5.4)	29 (5.9)	0.823
Sex (n, %, male)	169 (46.3)	60 (46.5)	229 (46.4)	0.567
Sleep (hours)	7.0 (1.1)	6.9 (1.2)	6.9 (1.1)	0.403
Smoker (current or former, n,%)	183 (50.1)	67 (51.9)	250 (50.6)	0.355
Stroke/TIA	18 (4.9)	4 (3.1)	22 (4.5)	0.156
Time to last follow-up (years)	3.9 (1.5)	3.9 (1.4)	3.9 (1.5)	0.870

Data are shown as mean (SD, range) if not otherwise specified. All data represent baseline data.

a Calculated as weight in kilograms divided by height in meters squared.

b Missing for 45 participants, 9.1 % of study sample.

### 2.5 Outcomes

For WMH the ratio between WMH and total cerebral volume (mm^3^) was used as outcome, composite temporal SUVr were used for tau PET and global Aβ PET accumulation was determined using the Centiloid Scale [30].

### 2.6 Statistical analysis

Demographic differences were tested for using the chi-square test (for binary/categorical variables) and the Mann-Whitney U test (for continuous variables) for group comparisons. Linear mixed-effects models with random intercepts and slopes were used to evaluate the associations between predictors and the longitudinal accumulation of WMH volume, Aβ, and tau in temporal ROIs (outcomes). A model incorporating interaction effects between time and the primary predictor adjusted for baseline age and sex was used in the linear mixed-effects models for all CU participants.

A sensitivity analysis for associations with tau accumulation included only CU Aβ+ individuals to better capture true tau accumulation in Aβ+ individuals and to reduce the risk of detecting tau signals that are likely attributed to noise.

All statistical analyses were performed using R version 4.2.1 (R Foundation for Statistical Computing). A *p*-value < .05 was considered to indicate statistical significance.

## 3 Results

### 3.1 Participants and baseline demographics

A total of 494 participants were included in the study, consisting of 365 CU Aβ− individuals and 129 CU Aβ+ individuals (Table 1). The average age was 64.8 (standard deviation, SD 13.3) years in the whole population, with the Aβ+ group being significantly older (mean 71.2, SD 8.9 years) than the Aβ− group (mean 62.5, SD 13.9 years) (*p* *<* *0.001*). The average follow-up time was 3.9 years (SD, 1.5 years) and did not differ between groups. A higher proportion of *APOE* ε4 carriers was observed in the Aβ+ group (77 %) compared to the Aβ− group (40 %) (*p* *<* *0.001*). Systolic blood pressure was significantly higher in the Aβ+ group (146 mmHg vs. 141 mmHg, *p* *=* *0.018*), while BMI was significantly lower (25.8 vs. 26.9, *p* *<* *0.001*). The Aβ+ individuals had higher prevalence of hyperlipidemia, hypertensive or cardioprotective drugs and diabetes (*p* *<* *0.05-<0.001*). The Aβ+ group had a higher burden of WMH on MRI, assessed using the Fazekas rating scale, with 21 % having a score of 2 and 15 % a score of 3, compared to 16 % and 4 % in the Aβ- group (*p* *<* *0.001*). The Aβ+ group also had a higher proportion of individuals living alone, 34 % compared to 27 % (*p* *<* *0.001*), and slightly higher depression score on the HADS, with a mean of 2.2 points and 2.1 points for the Aβ- group (*p* *<* *0.001*). Three Aβ+ individuals fulfilled criteria for probable CAA according to the Boston 2.0 criteria for CAA. No familial mutations known to increase the risk of AD are known among the participants.

### 3.2 Prediction of longitudinal WMH accumulation

**Fig. 1:**
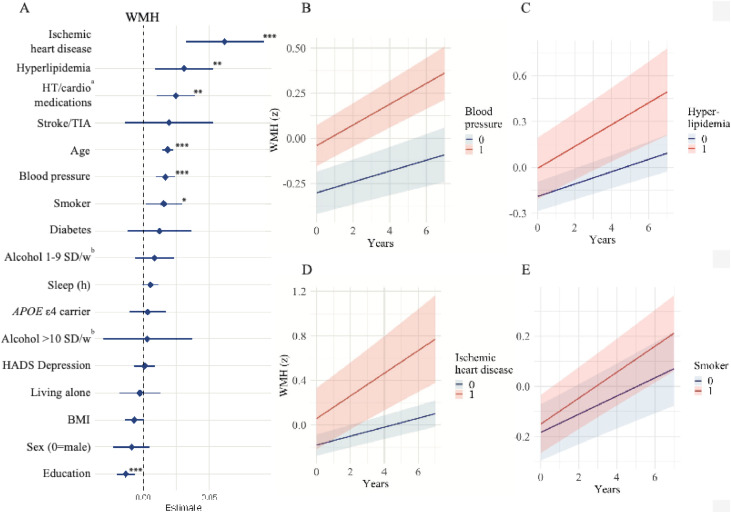
Forest plot of longitudinal WMH accumulation.

Associations between predictors and accumulation of WMH are presented in Fig. 1 and supplementary eTable 1. Significant predictors of longitudinal increase in WMH, adjusted for baseline age and sex, were higher age (β = 0.02, 95 % CI: 0.01–0.02; only adjusted for sex), higher blood pressure (β = 0.02, 95 % CI: 0.01–0.02), presence of hyperlipidemia (β = 0.03, 95 % CI: 0.01–0.05), and ischemic heart disease (β = 0.06, 95 % CI: 0.03–0.09), smoking (β = 0.02, 95 % CI: 0.00–0.03) and higher education (β = −0.01, 95 % CI: −0.02– −0.01). Numbers and % of participants with available MRI data at each follow-up visit are shown in the supplementary eTable 2.

A. Forest plot of predictors for white matter hyperintensities (WMH)/intracerebral volume ratio on standardized scales, ranked by effect estimate and 95 %CI. All continuous predictors where transformed to z-scores based on the distribution in the current population. Models incorporated interaction terms between time and the primary predictor of interest, while adjusting for baseline age and sex. Levels of significance: *<0.05, **p<0.01, **p<0.001.^a^Hypertension and cardioprotective medications. ^b^SD/w represents standard drinks/week. B. WMH accumulation stratified by systolic blood pressure, dichotomized at the median (142 mmHg); C. WMH accumulation stratified by presence of hyperlipidemia; D. WMH accumulation among individuals with prevalent ischemic heart disease at baseline; E. WMH accumulation among current or former smokers and non-smokers.

### 3.3 Prediction of longitudinal Aβ accumulation

**Fig. 2:**
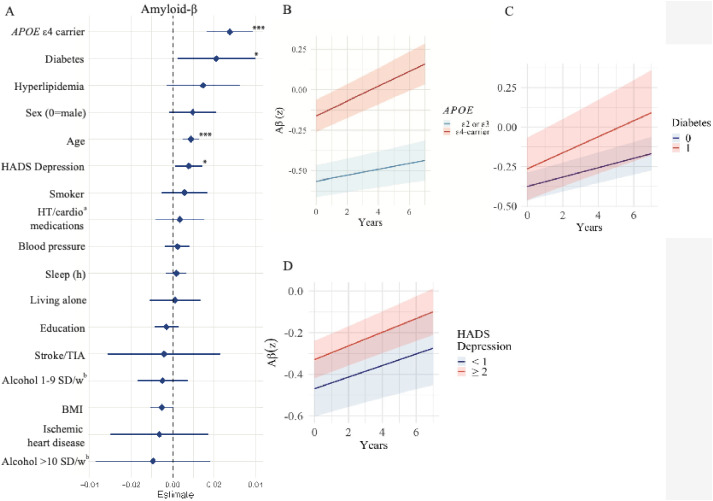
Forest plot of longitudinal Aβ accumulation.

Associations between modifiable and non-modifiable risk factors and longitudinal accumulation of Aβ are shown in Fig. 2 and supplementary eTable 3. Adjusted for baseline age and sex, significant predictors of faster accumulation of Aβ included higher age (β = 0.01, 95 % CI: 0.00–0.01; only adjusted for sex), being *APOE* ε4 carrier (β = 0.03, 95 % CI: 0.02–0.04), higher score on HADS Depression scale (β = 0.01, 95 % CI: 0.00–0.01) and diabetes (β = 0.02, 95 % CI: 0.00–0.04). Numbers and % of participants with available Aβ PET data at each follow-up visit are shown in the supplementary eTable 2.

A. Forest plot of predictors of amyloid-β (Aβ) accumulation on standardized scales, ranked by effect estimate and 95 %CI. All continuous predictors where transformed to z-scores based on the distribution in the current population. Models incorporated interaction terms between time and the primary predictor of interest, while adjusting for baseline age and sex. Levels of significance: *<0.05, **p<0.01, **p<0.001.^a^Hypertension and cardioprotective medications. ^b^SD/w represents standard drinks/week. B. Aβ accumulation stratified by *APOE* ε4 carrier status; C. Aβ accumulation stratified by diabetes at baseline. D. Aβ accumulation stratified by scores above or below the median on the HADS Depression subscale.

### 3.4 Prediction of longitudinal tau accumulation

**Fig. 3:**
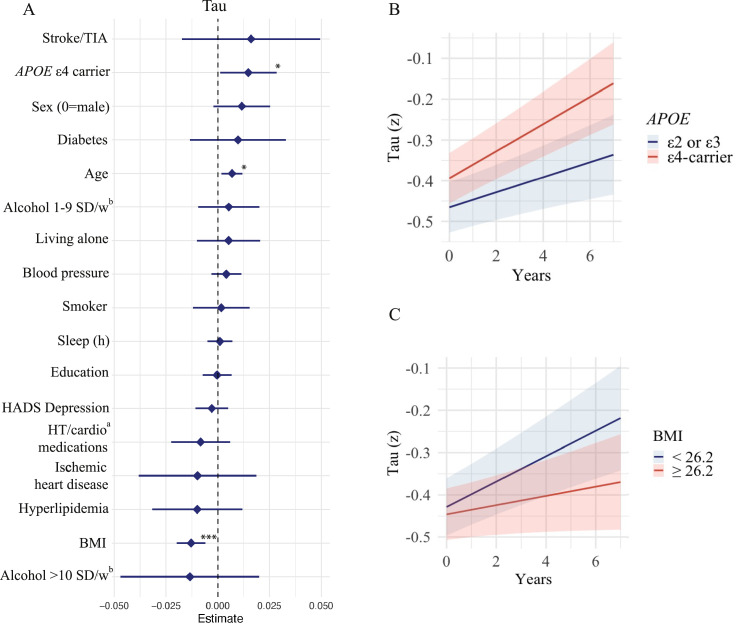
Forest plot of longitudinal tau accumulation.

Prediction of longitudinal tau accumulation is presented in Fig. 3 and supplementary 4. Higher age (β = 0.01, 95 % CI: 0.00–0.01; only adjusted for sex), *APOE* ε4 carrier (β = 0.01 95 % CI: 0.00–0.03) and lower BMI (β = −0.01 95 % CI: −0.02– −0.01) were significantly associated with higher tau accumulation, adjusted for baseline age and sex. See sensitivity analysis below for results in Aβ positive participants. Female sex only reached trend level of significance (β = 0.01 95 % CI: −0.00–0.03), adjusted for baseline age. Numbers and % of participants with available tau PET data at each follow-up visit are shown in the supplementary eTable 2.

A. Forest plot of predictors of tau accumulation, on standardizes scales ranked by effect estimate and 95 %CI. All continuous predictors where transformed to z-scores based on the distribution in the current population. Models incorporated interaction terms between time and the primary predictor of interest, while adjusting for baseline age and sex. Levels of significance: *<0.05, **p<0.01, **p<0.001.^a^Hypertension and cardioprotective medications. ^b^SD/w represents standard drinks/week. B. Aβ B. Tau accumulation stratified by *APOE* ε4 carrier status; C. Tau accumulation stratified by body mass index (BMI), dichotomized at the median (26.2 kg/m²).

### 3.5 Sensitivity analyses

To further explore the association between HADS Depression scores and increased Aβ accumulation, we adjusted for whether the participant had SCD or was cognitively healthy, based on a hypothesis that among individuals with SCD, subtle depressive symptoms could reflect early Aβ accumulation rather than constitute a risk factor. After this adjustment, HADS Depression was no longer significant.

We also examined the associations between modifiable and non-modifiable risk factors and tau accumulation in exclusively Aβ+ individuals, as this subpopulation is where the tau PET signal is most likely to reflect tau tangle pathology rather than measurement noise. The results are shown in Table 2. Only lower BMI was still associated with higher tau accumulation (β = −0.03 95 % CI: −0.05– −0.02). In Aβ+ individuals, age and *APOE* ε4 carrier status were no longer significant predictors of tau accumulation.

**Table 2:** Prediction of tau accumulation in Aβ+ participants.

	Multivariable β (95 % CI)
Age	0.01 (−0.01–0.03)
Alcohol	
0 (reference)	Ref
1–9 standard drinks/week	0.01 (−0.03–0.05)
>10 standard drinks/week	−0.06 (−0.15–0.03)
*APOE genotype*	
ε2 and/or ε3 (reference)	Ref
ε4-carrier	0.01 (−0.03–0.05)
Blood pressure, systolic	0.01 (−0.01–0.03)
**BMI**	−0.03 (−0.05– −0.02)***
Depression, HADS-scores	−0.01 (−0.03–0.01)
Diabetes	0.02 (−0.07–0.03)
Education	−0.01 (−0.01–0.02)
Hyperlipidemia	−0.02 (−0.07–0.03)
Hypertensive or cardioprotective medications	−0.02 (−0.06–0.01)
Ischemic heart disease	−0.02 (−0.09–0.05)
Living alone	0.01 (−0.05–0.02)
Sex, 0=male	0.02 (−0.01–0.05)
Sleep, hours/night	0.01 (−0.01–0.02)
Smoker, current or former	−0.02 (−0.05–0.01)
Stroke/TIA	0.03 (−0.07–0.12)

Results were obtained with linear mixed-effects models, with random intercepts and slopes, using longitudinal tau accumulation as outcome (standardized scales), in only Aβ + individuals (*n* = 129). Model included interaction between time and the predictor, with adjustment for baseline age and sex. All predictors represent baseline data and continuous variables on standardized scales.

Given the considerably smaller Aβ+ population, we additionally examined longitudinal tau accumulation in the entire study cohort, adjusting for baseline Aβ PET levels. The results were similar with those observed in the Aβ+ subgroup: lower BMI was significantly associated with increased tau accumulation, along with increasing age. *APOE* genotype was no longer significant (data not shown).

We further examined the association between low BMI and increased accumulation of tau, adjusting for cognitive status at baseline (i.e., control or SCD), and found that the association remained consistent.

## 4 Discussion

In this longitudinal prospective cohort study of 494 cognitively unimpaired individuals with on average four-year follow-up data, we found that known modifiable and non-modifiable risk factors of dementia [1] primarily modulated WMH, rather than Aβ and tau accumulation. Higher age was a consistent predictor of the accumulation of all three pathologies. Metabolic risk factors for cardiovascular disease such as high blood pressure, hyperlipidemia, presence of cardiovascular disease and smoking, were associated with a longitudinal increase of WMH. Diabetes was linked to greater Aβ accumulation. Presence of the *APOE* ε4 allele was linked to greater accumulation of both Aβ and tau, while lower BMI was only associated with increased tau accumulation. However, within Aβ+ individuals, only low BMI remained a significant predictor of tau accumulation, suggesting that *APOE* ε4 and age primarily are predictors of Aβ pathology which is a prerequisite of neocortical tau accumulation [31].

The main strengths of the study are the relatively large sample size for an AD biomarker study with at least two examinations of MRI, Aβ PET and tau PET, the prospective study design, and inclusion of only cognitively unimpaired individuals to reduce the risk of reverse causation (i.e., that the presence of a certain predictor would be caused by a manifest, symptomatic neurodegenerative disease). While previous studies have examined the longitudinal accumulation of WMH, Aβ, and tau separately, an important strength is to investigate both modifiable and non-modifiable risk factors in relation to all three pathologies in a head-to-head longitudinal comparison.

Cardiovascular risk factors are known to be associated with cerebrovascular pathology [32], and intervening on them has been shown to reduce cardiovascular risk [33]. An association was confirmed in our analyses, where higher blood pressure, hyperlipidemia, use of antihypertensive or cardioprotective medications, ischemic heart disease and smoking (current or former) were all significantly associated with increasing WMH over time. Chronic high blood pressure damages small arterioles through sustained elevated pressure, causing arteriolosclerosis, but venular collagenosis also contributes to cerebral small vessel disease [34]. Smoking contributes to cerebral small vessel disease and WMH through multiple pathological pathways. It causes endothelial damage through impaired nitric oxide-mediated endothelial function and promotes inflammation [35]. Intervening on these metabolic and vascular risk factors may therefore help reduce the burden of cerebral small vessel disease, as reflected by WMH. By addressing these modifiable risks, such interventions could not only slow the progression of brain vascular injury but also reduce the synergistic impact of co-existing neuropathologies, such as tau and Aβ accumulation. Ultimately, this may contribute to delaying cognitive decline and lowering the overall incidence of dementia in aging populations.

The strongest risk factor for Aβ accumulation was non-modifiable: carriership of the *APOE* ε4 allele, which is widely recognized as a risk factor for AD due to its association with higher Aβ accumulation. Also, a diagnosis of diabetes at baseline was linked to increased Aβ accumulation. Previous studies have shown conflicting results on diabetes and accumulation of Aβ and tau. One study reported higher Aβ accumulation in hippocampus but not other ROIs among MCI participants with diabetes, but not significant among CU individuals. They also found an increase of tau accumulation [36]. In contrast, other studies have found no relationship between HbA1c and Aβ-pathology [37], nor with type 2 diabetes and AD pathology [38]. Additionally, neuropathological studies have repeatedly shown no significant association between diabetes and AD pathology [[39], [40], [41], [42]]. Potential pathophysiological mechanisms include competition for degradation by shared enzymes such as insulin-degrading enzyme, which also regulates Aβ clearance. In addition, insulin resistance and reduced insulin-like growth factor (IGF-1) impair brain insulin signaling, thereby promoting Aβ accumulation. Dysregulated insulin signaling may further disrupt amyloid precursor protein (APP) metabolism, leading to enhanced Aβ production and aggregation [43]. This finding should be investigated further in future studies, but could be important regarding a potential role of novel diabetic treatments as a way of slowing cognitive decline in very early AD [44,45].

Depressive symptoms, assessed using the HADS depression scale, were significantly associated with Aβ accumulation. Nevertheless, this association was no longer significant after additional adjustment for baseline cognitive status (cognitively healthy control or SCD), suggesting that depressive symptoms may reflect an early manifestation of underlying pathology prior to objective cognitive impairment.

In this study, we found significant associations between *APOE* ε4 and faster tau accumulation. Though, when including only Aβ+ individuals or when adjusting for baseline Aβ PET SUVr, this association was no longer significant, indicating that the accelerated tau accumulation is primarily driven by Aβ accumulation. Findings indicating that the impact of the *APOE* ε4 allele on tau burden is primarily mediated by the predominant effect of Aβ pathology are consistent with previously published studies [46,47].

Previous studies have demonstrated that tau accumulates more rapidly in women compared to men [16,17,48]. In the present study, although a trend toward greater tau accumulation in females was observed, consistent with earlier findings, it did not meet the threshold for statistical significance. This may be explained by limited statistical power. The only modifiable risk factor for tau accumulation observed among Aβ+ individuals was lower BMI. This finding was consistent when adjusting for cognitive status at baseline (control or SCD). Low BMI has previously been shown to be associated with both higher Aβ and tau burden in one study [49], but in another only to greater tau accumulation [50]. Mid-life increased BMI is thought to be a risk factor for dementia but late-life increased BMI is protective [51,52]. It could be hypothesized that lower BMI could be associated with early change in dietary habits and reduced calorie intake among individuals with cognitive impairment. These dietary changes may then begin long before cognitive symptoms appear, suggesting that the association between lower BMI and tau accumulation could be driven by an early symptom of cognitive decline, even though the present study focused on CU individuals. An additional hypothesis is that the neurodegenerative itself causes increased resting energy expenditure already at preclinical stages. For example, data from AD transgenic mouse models showed an increased metabolic rate (24 % higher oxygen consumption and 29 % higher CO_2_ production) accompanied by weight-loss despite higher food consumption, compared to non-transgenic control mice [53]. Also, in a cohort with autosomal dominant AD, comprising preclinical mutation carriers and their non-carrier relatives as controls, the authors observed weight loss in mutation carriers as early as 20 years prior to symptom onset. They hypothesized that this may reflect early disturbances in central mechanisms regulating weight in AD [54]. Also, cognitive impairment in individuals who are overweight may arise from a more heterogeneous range of underlying pathophysiological mechanisms. In contrast, those with lower body mass index (BMI) tend to have a reduced risk of cerebral vascular pathology, likely due to a decreased prevalence of metabolic syndrome [55]. Consequently, the likelihood of multiple concurrent brain pathologies is also lower among individuals with lower BMI.

Low education is a well-established risk factor for dementia [1] and was, in this study, associated with greater accumulation of WMH, but not with Aβ or tau pathology. Education is linked to other risk factors such as socioeconomic status, healthy lifestyle [56], and smoking [57], which may underlie its association with WMH. The absence of associations with Aβ and tau suggests that low education, which in previous studies have been highlighted as risk factor for AD [58], may relate more strongly to cognitive reserve [59] than being a risk factor for AD pathology.

Finally, commonly discussed risk factors, including sleep duration, living alone, and alcohol consumption [60], were not associated with the accumulation of WMH, Aβ, or tau in this cohort.

This study has limitations. First, although we found robust associations, future similar studies may need longer follow-up periods than four years to detect subtle changes in these types of biomarkers. Second, this study did not include an independent validation cohort, and the results should preferably be replicated in other cohorts. Third, the current population was predominantly white, which means we cannot draw conclusions about differences or effects in other ethnic groups. Fourth, we have not adjusted for potential medication use, as possible confounder, which can be a limitation. Fifth, socioeconomic status has been shown to be associated with a higher prevalence of hypertension, smoking, diabetes and obesity, which in turn may influence associations with WMH [1]. We do not have information on socioeconomic status, nor on information on social activities, hobbies, or physical activity, all of which would have been valuable to examine. Last, as with all non-randomized observational studies, the ability to establish causal relationships is limited, and the possibility of residual confounding cannot be fully excluded, despite adjustment for potential confounding and mediating factors at baseline.

## 5 Conclusion

Modifiable risk factors of future dementia primarily affect accumulation of cerebral vascular pathology, rather than Aβ and tau. Carriage of the *APOE* ε4 allele or having diabetes was linked to greater Aβ accumulation, while lower BMI was associated with higher accumulation of tau. Further studies are needed to understand the mechanisms underlying the potential associations between diabetes and Aβ accumulation as well as lower BMI and tau accumulation.

## Funding

Work at the authors’ research group was supported by the National Institute of Aging (#R01AG083740), the Alzheimer’s Association (SG-23–1,061717, ALZSI-26–1,523522, ZEN24–1,069572), European Research Council (ADG-101096455), GHR Foundation, Swedish Research Council (2022-00775, 2021-00905, 2018-02052, 2021-02219, and 2017-01541), ERA PerMed (ERAPERMED2021–184), the Knut and Alice Wallenberg foundation (2017-0383, 2022-0231), the Strategic Research Area MultiPark (Multidisciplinary Research in Parkinson's disease) at Lund University, the Swedish Alzheimer Foundation (AF-981132, AF-980907 and AF-968598, AF-1,011949, AF-1,011799), the Swedish Brain Foundation (FO2021–0293, FO2022–0204, FO2023–0163, FO2024–0284, FO2025–0055), The Michael J Fox Foundation (MJFF-025507), Lilly Research Award Program, Greta and Johan Kock Foundation, WASP and DDLS Joint call for research projects (WASP/DDLS22–066), The Parkinson foundation of Sweden (1280/20 and 1412/22), the Kamprad Foundation (20243058), the Cure Alzheimer's fund, the Konung Gustaf V:s and Drottning Victorias Frimurarestiftelse, Bundy Academy, Rönström Family Foundation, the Skåne University Hospital Foundation (2020-O000028), Regionalt Forskningsstöd (2022-1259 and 2022-1346) and the Swedish federal government under the ALF agreement (2022-Projekt0080, 2022-Projekt0085, 2018-Projekt0279, 2022-Projekt0107 and 2021-ST0019).

Doses of ^18^F-flutemetamol injection were sponsored by GE Healthcare. The precursor of ^18^F-flortaucipir was provided by AVID radiopharmaceuticals. The precursor of ^18^F-flutemetamol was sponsored by GE Healthcare. The precursor of ^18^F-RO948 was provided by Roche.

The funding sources had no role in the design and conduct of the study; in the collection, analysis, interpretation of the data; or in the preparation, review, or approval of the manuscript.

## Disclosures

I.G. has nothing to disclose. N.MC. has received consultancy/speaker fees from Biogen, Eli Lilly, Owkin and Merck. E.S. has acquired research support (for the institution) from C2N Diagnostics, Fujirebio, GE Healthcare and Roche Diagnostics. R.O. has received research funding/support from Avid Radiopharmaceuticals, Janssen Research & Development, Roche, Quanterix and Optina Diagnostics, has given lectures in symposia sponsored by GE Healthcare, received speaker fees from Springer, is an advisory board/steering committee member for Asceneuron, Biogen, Johnson & Johnson and Bristol Myers Squibb. All the aforementioned has been paid to his institutions. D.vW. has nothing to disclose. O.H. is an employee of Lund University and Eli Lilly. S.P. has acquired research support (for the institution) from Avid and ki elements through ADDF. In the past 2 years, he has received consultancy/speaker fees from Bioartic, Biogen, Eisai, Eli Lilly, Novo Nordisk, and Roche.

## Declaration of generative AI and AI-assisted technologies in the writing process

During the preparation of this work the authors used ChatGPT to improve and find errors in scripts in R. After using this tool/service, the authors reviewed and edited the content as needed and take full responsibility for the content of the publication.

## CRediT authorship contribution statement

**Isabelle Glans:** Writing – original draft, Visualization, Project administration, Funding acquisition, Formal analysis, Conceptualization. **Niklas Mattsson-Carlgren:** Writing – review & editing, Supervision. **Olof Strandberg:** Data curation. **Erik Stomrud:** Writing – review & editing, Project administration. **Rik Ossenkoppele:** Writing – review & editing. **Danielle van Westen:** Writing – review & editing, Investigation. **Nicola Spotorno:** Writing – review & editing. **Oskar Hansson:** Writing – review & editing. **Sebastian Palmqvist:** Writing – review & editing, Supervision, Conceptualization.

## Declaration of competing interest

The authors declare the following financial interests/personal relationships which may be considered as potential competing interests: Oskar Hansson reports financial support was provided by National Institute on Aging. Oskar Hansson reports financial support was provided by Alzheimer’s Association. Oskar Hansson reports financial support was provided by European Research Council. Oskar Hansson reports financial support was provided by GHR Foundation. Oskar Hansson reports financial support was provided by Swedish Research Council. Oskar Hansson reports financial support was provided by Knut and Alice Wallenberg Foundation. Oskar Hansson reports financial support was provided by Strategic Research Area Multipark. Oskar Hansson reports financial support was provided by Swedish Alzheimers Foundation. Oskar Hansson reports financial support was provided by Swedish Brain Foundation. Oskar Hansson reports financial support was provided by The Michael J Fox Foundation. Oskar Hansson reports financial support was provided by ERA PerMed. Oskar Hansson reports financial support was provided by Lilly Research Award Program. Oskar Hansson reports financial support was provided by Greta and Johan Kock Foundations. Oskar Hansson reports financial support was provided by WASP and DDLS Joint calls for research projects. Oskar Hansson reports financial support was provided by The Parkinsons foundation of Sweden. Oskar Hansson reports financial support was provided by The Kamprad Family Foundation. Oskar Hansson reports financial support was provided by Cure Alzheimer’s Fund. Oskar Hansson reports financial support was provided by King Gustaf V and Queen Victoria’s Masonic Foundation. Oskar Hansson reports financial support was provided by Bundy Academy Foundation. Oskar Hansson reports financial support was provided by Rönström Family Foundation. Oskar Hansson reports financial support was provided by Skåne University Hospital. Oskar Hansson reports financial support was provided by Regionalt forskningsstöd. Isabelle Glans reports financial support was provided by Swedish federal government under the ALF-agreement. Niklas Mattsson-Carlgren reports a relationship with Biogen that includes: consulting or advisory and speaking and lecture fees. Niklas Mattson-Carlgren reports a relationship with Eli Lilly that includes: consulting or advisory and speaking and lecture fees. Niklas Mattson-Carlgren reports a relationship with Owkin Inc that includes: consulting or advisory and speaking and lecture fees. Niklas Mattson-Carlgren reports a relationship with Merck & Co Inc that includes: consulting or advisory and speaking and lecture fees. Erik Stomrud reports a relationship with C2N Diagnostics, LLC that includes: funding grants. Erik Stomrud reports a relationship with FUJIREBIO Inc that includes: funding grants. Erik Stomrud reports a relationship with GE Healthcare that includes: funding grants. Erik Stomrud reports a relationship with Roche Diagnostics that includes: funding grants. Rik Ossenkoppele reports a relationship with Avid Radiopharmaceuticals Inc that includes: funding grants. Rik Ossenkoppele reports a relationship with Janssen Research and Development that includes: funding grants. Rik Ossenkoppele reports a relationship with Roche that includes: funding grants. Rik Ossenkoppele reports a relationship with Quanterix Corp that includes: funding grants. Rik Ossenkoppele reports a relationship with Optina Diagnostics that includes: funding grants. Rik Ossenkoppele reports a relationship with GE Healthcare that includes: speaking and lecture fees. Rik Ossenkoppele reports a relationship with Springer that includes: speaking and lecture fees. Rik Ossenkoppele reports a relationship with Asceneuron SA that includes: board membership. Rik Ossenkoppele reports a relationship with Biogen that includes: board membership. Rik Ossenkoppele reports a relationship with Johnson & Johnson that includes: board membership. Rik Ossenkoppele reports a relationship with Bristol Myers Squibb Co that includes: board membership. Oskar Hansson reports a relationship with Eli Lilly that includes: employment. Sebastian Palmqvist reports a relationship with Avid that includes: funding grants. Sebastian Palmqvist reports a relationship with Alzheimer’s Drug Discovery Foundation that includes: funding grants. Sebastian Palmqvist reports a relationship with BioArctic AB that includes: speaking and lecture fees. Sebastian Palmqvist reports a relationship with Biogen that includes: speaking and lecture fees. Sebastian Palmqvist reports a relationship with Eisai that includes: speaking and lecture fees. Sebastian Palmqvist reports a relationship with Eli Lilly that includes: speaking and lecture fees. Sebastian Palmqvist reports a relationship with Novo Nordisk that includes: speaking and lecture fees. Sebastian Palmqvist reports a relationship with Roche that includes: speaking and lecture fees. If there are other authors, they declare that they have no known competing financial interests or personal relationships that could have appeared to influence the work reported in this paper.

## Appendix Supplementary materials

Image, application 1
