# Supplementary material for: Associations of modifiable and non-modifiable risk factors with longitudinal white matter hyperintensities, amyloid-β and tau - a prospective cohort study
Source: J Prev Alzheimers Dis. 2026 Jan 1;13(2):100448. doi: 10.1016/j.tjpad.2025.100448 (PMC12869030; doi:10.1016/j.tjpad.2025.100448)
Supplement: Supplementary file 1 [file mmc1.docx]

**Supplementary Material**

**Supplementary eMethods**

**Diagnostic and inclusion criteria**

BioFINDER-2 cohort A and B includes neurologically and cognitively healthy controls. The inclusion criteria are: i) ages 40-65 years (cohort A) and ages 66-100 years (cohort B); ii) absence of cognitive symptoms as assessed by a physician specialized in cognitive disorders; iii) MMSE score 27-30 (A) or 26-30 (cohort B) at screening visit; iv) do not fulfill the criteria for mild or major neurocognitive disorder (MCI or dementia) according to DSM-55; and v) fluent in Swedish. The recruitment process of cohorts A and B is designed to build two study populations with 50% *APOE* ε4 carriers in each. Cohort C comprises participants with subjective cognitive decline (SCD) or MCI. Inclusion criteria are: i) ages 40-100 years; ii) referred to the memory clinics due to cognitive symptoms; iii) MMSE score of 24-30 points; iv) does not fulfill the criteria for any dementia (major neurocognitive disorder) according to DSM-55, v) fluent in Swedish. In accordance with the research framework by the National Institute on Aging-Alzheimer’s Association, study participants with SCD were analyzed together with the cognitively healthy participants (and combined in the cognitively unimpaired [CU] group). Participants were classified as having MCI if they performed worse than -1.5 SD in any cognitive domain according to age and education stratified test norms. The neuropsychological battery covered the domains attention (Trail Making Test A and Symbol Digit Modalities Test), executive function (Trail Making B and A Quick Test of cognitive speed [AQT]), verbal ability (verbal fluency animals and the 15 word short version of the Boston Naming Test), memory (immediate and delayed recall from the Alzheimer’s Disease Assessment Scale [ADAS]), and visuospatial function (incomplete letters and cube analysis from the Visual Object and Space Perception battery [VOSP]). Those that were not classified as MCI were considered to have SCD.

**Alcohol consumption**

The questionnaire asks the participant to estimate how many standard drinks that are on average consumed during an ordinary week. One standard drink is defined as 33 cl of beer or cider (4.5% alcohol by volume), 12–15 cl of wine, or 4 cl of spirits (40% alcohol by volume).

**MRI protocol**

Participants underwent MRI scans on a Siemens MAGNETOM Prisma 3T scanner (Siemens Healthineers, Erlangen, Germany) equipped with a 64-channel head coil. A T1w MPRAGE sequence (TR = 1900 ms TE = 2.54 ms, in-plane resolution = 1 × 1 mm^2^, slice thickness = 1 mm) was acquired as well as a T2-weighted FLAIR scan (TR = 5000 ms, TE = 393 ms, same resolution and FOV as for the MPRAGE).

**Supplementary eTables**

**eTable1. Prediction of WMH accumulation.**

|  | β (95% CI) |
| --- | --- |
| **Age** | **0.02 (0.01**–**0.02)***** |
| Alcohol  0 (reference)  1-9 standard drinks/week  >10 standard drinks/week | 0.01 (-0.01–0.02)  -0.00 (-0.03–0.04) |
| *APOE genotype*  ε2 and/or ε3 (reference)  ε4-carrier | Ref  0.00 (-0.01-0.02) |
| **Blood pressure, systolic** | **0.02 (0.01–0.02)***** |
| BMI | -0.01 (-0.01- 0.00) |
| Depression, HADS-scores | 0.00(-0.01–0.01) |
| Diabetes | 0.01 (-0.01–0.04) |
| **Education** | **-0.01 (-0.02– -0.01)***** |
| **Hyperlipidemia** | **0.03 (0.01–0.05)**** |
| **Hypertensive or cardioprotective medication)** | **0.02 (0.01–0.04)**** |
| **Ischemic heart disease** | **0.06 (0.03-0.09)***** |
| Living alone | -0.01 (-0.02–0.01) |
| Sex, 0=male | -0.01 (-0.02– -0.00) |
| Sleep, hours/night | 0.01 (-0.00-0.01) |
| **Smoker, current or former** | **0.02 (0.00–0.03)*** |
| Stroke/TIA | 0.02 (-0.01–0.05) |

Results were obtained with linear mixed models, with random intercepts and slopes, using longitudinal WMH/intracerebral volume ratio as outcome (standardized scales). Models included interaction between time and the predictor with adjustment for baseline age and sex. All predictors represent baseline data and continuous variables on standardized scales.

**eTable 2. Examinations during follow-up.**

| Visit (year from baseline) | MRI | Aβ PET | Tau PET |
| --- | --- | --- | --- |
| 0 (n, %) | 491 (99.4) | 489 (99.0) | 494 (100) |
| 2 (n, %) | 454 (91.9) | 452 (91.5) | 456 (92.3) |
| 4 (n, %) | 282 (57.1) | 194 (39.3) | 210 (42.5) |
| 6 (n, %) | 42 (8.5) | 39 (7.9) | 38 (7.7) |

**eTable3. Prediction of Aβ accumulation.**

|  | β (95% CI) |
| --- | --- |
| **Age** | **0.01 (0.00**–**0.01)***** |
| Alcohol  0 (reference)  1-9 standard drinks/week  >10 standard drinks/week | Ref  -0.00 (-0.02–0.01)  -0.01 (-0.04–0.02) |
| *APOE-genotype*  ε2 and/or ε3 (reference)  **ε4-carrier** | **Ref**  **0.03 (0.02**–**0.04)***** |
| Blood pressure, systolic | 0.00 (-0.00–0.01) |
| BMI | -0.01 (-0.01–0.00) |
| **Depression, HADS-scores** | **0.01 (0.00–0.01)*** |
| **Diabetes** | **0.02 (0.00–0.04)*** |
| Education | -0.00 (-0.01–0.00) |
| Hyperlipidemia | 0.01 (-0.00–0.03) |
| Hypertensive or  cardioprotective medications | 0.00 (-0.01–0.02) |
| Ischemic heart disease | -0.01 (-0.03–0.02) |
| Living alone | 0.00 (-0.01–0.01) |
| Sex, (0=male) | 0.01 (-0.00-0.02) |
| Sleep, hours/night | 0.00 (-0.00–0.01) |
| Smoker, current or former | 0.01 (-0.01–0.02) |
| Stroke/TIA | -0.00 (-0.03–0.02) |

Results were obtained with linear mixed models, with random intercepts and slopes, using longitudinal Aβ accumulation as outcome (standardized scales). Models included interaction between time and the predictor with adjustment for baseline age and sex. All predictors represent baseline data and continuous variables on standardized scales.

**eTable 4. Prediction of tau accumulation.**

|  | β (95% CI) |
| --- | --- |
| **Age** | **0.01 (0.00**–**0.01)*** |
| Alcohol  0 (reference)  1-9 standard drinks/week  >10 standard drinks/week | Ref  0.01 (-0.01–0.02) -0.01 (-0.05–0.02) |
| *APOE-genotype*  ε2 and/or ε3 (reference)  **ε4-carrier** | Ref  **0.01 (0.00**–**0.03)*** |
| Blood pressure, systolic | 0.00 (-0.01–0.01) |
| **BMI** | **-0.01 (-0.02**– **-0.01)***** |
| Depression, HADS-scores | -0.00 (-0.01–0.00) |
| Diabetes | 0.01 (-0.01–0.03) |
| Education | -0.00 (-0.01–0.01) |
| Hyperlipidemia | -0.01 (-0.03–0.01) |
| Hypertensive or  cardioprotective medications | -0.01 (-0.02–0.01) |
| Ischemic heart disease | -0.01 (-0.04–0.02) |
| Living alone | 0.01 (-0.01–0.02) |
| Sex, 0=male | 0.01 (-0.00–0.03) |
| Sleep, hours/night | 0.00 (-0.00–0.01) |
| Smoker, current or former | 0.00 (-0.01–0.02) |
| Stroke/TIA | 0.01 (-0.02–0.05) |

Results were obtained with linear mixed models, with random intercepts and slopes, using longitudinal tau accumulation as outcome (standardized scales). Models included interaction between time and the predictor, with adjustment for baseline age and sex. All predictors represent baseline data and continuous variables on standardized scales.
